# Cost-consequence analysis of a digital health-enabled non-communicable disease management intervention in Ghana

**DOI:** 10.1186/s12962-026-00733-0

**Published:** 2026-02-27

**Authors:** Appiah Akwasi Obeng, Richard Abeiku Bonney, Thomas Yaw Ayensu Essel, Paulina Afia Gyinae Wilberforce, Peter Agyei-Baffour

**Affiliations:** 1 Department of Health Policy, Management and Economics, Kwame Nkrumah University of Science and Technology, Kumasi, Ghana; 2 Department of Health Care Management, Technische Universität Berlin, Berlin, Germany; 3 Department of Computer Science, Kwame Nkrumah University of Science and Technology, Kumasi, Ghana

**Keywords:** Cost-consequence analysis, Digital health, Non-communicable disease, Akoma Pa, Ghana

## Abstract

**Background:**

Diabetes and hypertension pose a major non-communicable disease (NCD) burden in Ghana, yet care remains reactive and resource-intensive. Although digital health interventions may improve screening, continuity of care, and patient engagement, economic evidence, particularly transparent assessments of multiple costs and outcomes, remains limited in low- and middle-income settings. The Akoma Pa initiative integrates digital health tools to address these gaps. There is a lack of evidence on the cost-consequences of digital health interventions in Ghana. This study evaluates the costs and consequences of the intervention compared with conventional standard care to inform resource allocation and policy decisions for sustainable NCD management in Ghana.

**Methods:**

A retrospective cost-consequence analysis was conducted comparing the Akoma Pa digital health program with conventional care for diabetes and hypertension in Christian Health Association of Ghana (CHAG) facilities between January and December 2023. A total of 705 adults with hypertension and/or diabetes were systematically sampled from 16 facilities (8 intervention, 8 comparator) using proportionate-to-size allocation and random selection from clinic registers; only consenting participants who completed follow-up were analysed. Costs were disaggregated into capital, recurrent, personnel, and training categories. Consequences were reported as a profile of outcomes, including clinical changes (HbA1c, systolic blood pressure, body mass index) and process indicators (follow-up rates).

**Results:**

The total annual program cost for the intervention was $508,443.89, compared with $529,882.28 for the comparator, indicating a cost-saving of approximately $21,438. The intervention demonstrated superior clinical effectiveness with greater mean reductions in HbA1c (-2.10% vs. -1.68%), Systolic Blood Pressure (-54.38 mmHg vs. -51.88 mmHg), and BMI (-4.42 kg/m² vs. -0.61 kg/m²). Process outcomes revealed a significant impact on engagement, with the intervention achieving an 89% follow-up rate compared to 36.5% in standard care.

**Conclusion:**

The Akoma Pa intervention presents a dominant economic profile, offering superior clinical and engagement outcomes at a lower total health system cost than conventional care. The efficiency gains from digital supply chain management and tele-counselling support the integration of such digital ecosystems into national NCD strategies.

## Background

Diabetes and hypertension are the most prevalent non-communicable diseases (NCDs) in Ghana, significantly contributing to the country’s health burden [1]. According to a World Health Organization (WHO) report, 3.2 million Ghanaians aged 30–79 years had been diagnosed with hypertension by 2019, with only 19% controlled [2]. A recent scoping review found the national-level prevalence of diabetes in Ghana ranging from 2.80% to 3.95% [3]. Ghana Health Service records an average of 200,000 cases of diabetes reported to health facilities annually [4]. This trend is expected to continue. These figures highlight the pressing need for effective strategies to manage NCDs in Ghana. The economic burden of diabetes and hypertension in Ghana extends beyond healthcare, significantly impacting households and the broader economy. Studies conducted in Ghana reveal that managing these chronic conditions leads to substantial out-of-pocket expenditures, worsening financial risk protection for families [5, 6]. A study by Togoe [7] reports that households with members living with NCDs face 49% higher healthcare costs compared with healthier households in Ghana. Another study conducted in the Eastern region of Ghana shows that the average monthly healthcare management cost for type 2 diabetes patients with complications is estimated at US$38.68, with direct costs constituting 94% of total expenses [8].

Conventional approaches to NCD management often focus on reactive care, such as glucose regulation, insulin control, and medication adherence [9]. While these strategies provide some benefits, they may fall short of addressing the broader metabolic and psychosocial challenges associated with NCDs [10, 11]. Moreover, these methods rely heavily on paper-based records, in-person consultations, and patient self-discipline, which can be particularly challenging in resource-limited settings like Ghana. Barriers such as frequent hospital visits, high healthcare costs, inefficiencies, ineffectiveness, low value for money, delay in care delivery, and difficulty adhering to prescribed lifestyle changes further complicate NCD management, have also been reported in Ghana [12].

Digital health technologies offer a promising alternative to conventional approaches. These technologies can enhance NCD management by reducing costs through fewer complications, hospitalizations, and unnecessary consultations; improving adherence through personalized feedback, reminders, and educational materials; and expanding access to care, particularly in remote areas [13]. Beyond clinical management, digital health can promote health literacy and empower patients to take active roles in their care [14]. Also, digital health strategies offer significant economic advantages in healthcare. They can lead to cost savings through streamlined processes and optimized resource allocation [15]. However, despite these potential benefits, questions remain regarding the cost-consequences of these interventions compared to conventional methods.

Cost-consequence analysis (CCA) is a valuable approach in healthcare decision-making by presenting costs and outcomes in a disaggregated, tabular format that is more accessible to decision-makers than traditional cost-effectiveness ratios [16]. Unlike cost-utility analysis, CCA does not combine benefits and costs into a single ratio; instead, it provides a clear descriptive summary that allows decision-makers to form their own judgments about the relative importance of different outcomes [17]. This transparency enables decision-makers to select components most relevant to their perspectives and builds confidence in data credibility for resource allocation decisions [16]. CCA supports systematic decision-making under uncertainty, complementing other analytical approaches in complex healthcare environments [18].

This study contributes directly to the achievement of Sustainable Development Goal (SDG) 3.4, which aims to reduce premature mortality from NCDs through prevention and treatment and the promotion of mental health and well-being by 2030. Diabetes and hypertension are major drivers of NCD-related morbidity and mortality in Ghana [1], and strengthening their long-term management is critical to achieving this target. By evaluating the costs and multiple clinical and service delivery consequences of a digitally enabled care model for NCD management, this study provides evidence on how health system innovations can improve disease control, patient follow-up, and resource use efficiency. The findings offer policymakers practical insights for scalable approaches that can enhance continuity of care and strengthen health system responses to NCDs, thereby supporting national and global efforts toward SDG 3.4.

The need for effective and sustainable healthcare solutions is underscored by Ghana’s challenges with systemic health funding. Health sector allocation as a percentage of government expenditure has declined from 8.1% in 2019 to a projected 6.4% in 2025, which is below the Abuja Declaration target of 15% [19]. Volatile donor funding and reduced contributions from other revenues exacerbate these challenges, leaving Ghana’s health spending as a percentage of GDP below the lower-middle-income country average threshold of 2.3%. This financial instability highlights the importance of cost-effective interventions that can optimize limited resources while improving health outcomes.

In Ghana, a previous study has shown that telemedicine can reduce costs in acute care and avoid unnecessary referrals [20], but little evidence exists on the broader costs and multiple health system consequences of digital health interventions for long-term NCD management. Most prior evaluations have relied on cost-effectiveness methods that do not capture the range of clinical and service delivery outcomes relevant to chronic care. This study fills that gap by applying a cost-consequence analysis to assess how a digitally enabled NCD program affects both health outcomes and resource use within routine health system settings, providing novel evidence for policymakers in a resource-constrained context. This study, therefore, aims to conduct a cost-consequence analysis of Akoma Pa, a digital health-enabled NCD management intervention versus conventional NCD management to inform decision-making on digital health interventions in Ghana.

## Methods

### Study design and analytical framework

This study employed a retrospective cohort design within a Cost-Consequence Analysis (CCA) framework to evaluate the Akoma Pa digital health program relative to standard NCD care. A CCA approach was selected because the intervention generates multiple clinical and service delivery outcomes that cannot be meaningfully reduced to a single summary effectiveness measure. This approach separates costs and consequences, allowing decision-makers to transparently assess trade-offs between investment (e.g., technology costs) and outcomes (e.g., clinical control and patient retention) and aligns with the Consolidated Health Economic Evaluation Reporting Standards (CHEERS 2022). The analysis covered a 12-month time horizon from January to December 2023 and compared two cohorts: eight facilities implementing the Akoma Pa program (intervention arm) and eight matched facilities providing routine NCD care without digital components (comparator arm).

### Study setting

The research was conducted in Ghana’s Central Region, which is characterized by a predominantly rural population and a high reliance on agriculture [21]. Facilities participating in the Akoma Pa program were purposively selected for inclusion in the intervention arm. Comparator facilities were selected and matched based on service level, patient volume, and geographical characteristics to enhance comparability between the two groups and reduce contextual differences that could influence outcomes.

### Study population and sampling

The study population consisted of adult patients aged 18 years and above with a confirmed diagnosis of hypertension and/or diabetes mellitus who were receiving care at the selected health facilities. A systematic random sampling approach was used to select patients from 16 healthcare facilities, comprising eight (8) facilities implementing the Akoma Pa intervention and eight (8) comparator facilities providing standard care. A total of 705 participants were included, with 367 patients from intervention facilities and 338 from comparator facilities. Proportionate-to-size sampling was applied by allocating the number of participants selected from each facility based on the relative number of eligible patients recorded in facility registers. Within each facility, patients were randomly selected during clinic days from the sampling frame to ensure an equal probability of selection (see Table 1). Participants who consented were included in the study. Patients who were unable to complete follow-up assessments due to severe complications were excluded from the analysis.

**Table 1:** Sample of study sites

Study sites	Patients enrolled	Estimated sample size	Participants (*n* = 367)
**Intervention sites**			
Baa Salvation Army Clinic, Ajumako	639	7.94%	29
Mercy Women’s Hospital, Mankessim	1216	15.12%	55
Our Lady of Grace Hospital, Assin Fosu	1288	16.01%	59
Presbyterian Health Centre, Assin Nsuta	526	6.54%	24
Salvation Army Hospital, Agona-Duakwa	885	11.00%	40
St. Gregory Catholic Hospital, Buduburam	1151	14.31%	53
St. Francis Xavier Hospital, Foso	1121	13.94%	51
St. Luke Catholic Hospital, Apam	720	8.95%	33
**Comparator sites**			**(** ***n*** ** = 338)**
Pentecost Hospital, Ayanfuri	503	18.06%	61
SDA Clinic, Ayanfuri	189	6.79%	23
Ochiso Salvation Army Health Centre, Ajumako	15	0.54%	2
Assin Praso Presby Hospital, Assin North	1765	63.38%	214
Infant Jesus Catholic Clinic, Kasoa North	97	3.48%	12
Siloam Mission Hospital, Kasoa Main	20	0.72%	2
Agona Pentecost Clinic, Morkwa	178	6.39%	22
Abrafo Pentecost Clinic, Fram	18	0.65%	2

### Description of the Akoma Pa intervention

The Akoma Pa program, launched in 2021 by CHAG, Novartis, Medtronic LABS, and GIZ, aims to address critical gaps in conventional NCD care by expanding access to integrated diabetes and hypertension management across 85 faith-based healthcare facilities in nine regions of Ghana [22]. The program targets low-income and underserved populations (e.g., aged, pensioners, farmers, etc.), enhancing early screening, clinical management, and patient support through digital health solutions and capacity-building for 2,231 healthcare professionals. Medtronic LABS supports digital patient tracking and remote monitoring, while Novartis SSA provides fully subsidized medications, reducing financial barriers to care [22]. Eight (8) health facilities that participated in the Akoma Pa program in the Central Region were purposively selected, with the remaining facilities serving as control sites for comparative analysis.

The study compared the Akoma Pa intervention, an integrated diabetes and hypertension management initiative, with standard care provided, a reactive approach initiated by patient presentation, with no systematic remote follow-up. The Akoma Pa program included tele-counselling, prescription refill reminders, medication adherence counselling, and patient support groups, which were absent in standard care. The comparator was selected to evaluate the clinical and cost benefits of the Akoma Pa intervention versus conventional care.

### Costing methodology

Costs were estimated from a healthcare system perspective and organized into capital, recurrent, and training and implementation cost categories, consistent with program costing approaches for digital health interventions. All costs were collected in Ghana Cedis and converted into United States Dollars using the average 2023 exchange rate of 1 USD to 11.01 GHS.

Capital costs included the amortized value of tablets, mobile phones, blood pressure monitors, weighing scales, and server infrastructure required for digital operations. Useful life assumptions were applied to annualize these costs, such as three years for mobile phones and five years for computers and related equipment. Recurrent costs captured personnel salaries for both routine facility staff and program-specific staff, utilities, internet data, maintenance, and medical consumables, including test strips and lancets. Training and implementation costs included expenses related to initial training workshops, train-the-trainer sessions, and Community Health Volunteers (CHV) sensitization and onboarding activities necessary for effective program delivery.

### Measurement of consequences

In line with CCA principles, consequences were measured across clinical and service delivery domains and reported separately from cost estimates. Clinical outcomes were assessed by measuring mean changes from baseline to month 12 in systolic blood pressure, glycated haemoglobin (HbA1c), fasting blood sugar, and body mass index. These indicators were selected because they reflect meaningful improvements in diabetes and hypertension management.

Process outcomes focused on patient follow-up rates, defined as the proportion of scheduled visits completed within the study period. Clinical data for the intervention arm were extracted from electronic health records within the digital platform, while data for the comparator arm were obtained from facility registers. Additional data were collected using the Kobo Toolbox to ensure completeness and consistency.

### Data analysis

Descriptive statistical methods were used to summarize sociodemographic characteristics and baseline clinical indicators for both cohorts. Mean changes in clinical indicators and follow-up rates over the 12 months were calculated and compared between intervention and comparator groups. Costs and consequences were not combined into a single summary metric. Instead, findings were presented in a cost-consequence framework that displays total and per-patient costs alongside clinical and process outcomes. This presentation allows policymakers and program planners to assess the value of the Akoma Pa program across multiple dimensions relevant to health system performance.

### Sensitivity analysis

To assess the robustness of the cost estimates, one-way sensitivity analyses were conducted on key cost parameters. These included variations in useful life assumptions for capital items, changes in personnel cost allocations, and alternative assumptions regarding the distribution of training and implementation costs. This analysis tested the stability of cost estimates under plausible alternative scenarios.

### Ethics and consent

Ethical approval for the study was obtained from the Committee on Human Research Publication and Ethics (CHRPE) at Kwame Nkrumah University of Science and Technology (KNUST). The study adhered to the principles of the Helsinki Declaration, and all participants provided informed consent. Confidentiality and anonymity were maintained throughout the research process.

## Results

### Sociodemographic characteristics of patients

The study included 367 patients from the Akoma Pa intervention sites and 338 patients from the comparator CHAG sites. The average age of patients at the Akoma Pa facilities was 53 years, with 25% aged over 65, compared to an average age of 61 years at the comparator sites, where 42% were over 65. The majority of patients were female at both sites (51% at Akoma Pa and 77% at comparator sites). Employment status varied, with 47% of Akoma Pa patients employed, compared to 51% retired at the comparator sites. Educational attainment was higher in the comparator group, with 48% having completed tertiary education, compared to 28% at Akoma Pa facilities (see Table 2).

**Table 2:** Sociodemographic characteristics of patients

Variable	Akoma Pa *n* = 367 (%)	Conventional *n* = 338 (%)
Age (years)	53 (29, 79)	61 (30, 90)
24–35	55 (15)	35 (10)
36–45	68 (19)	28 (8)
46–55	74 (20)	62 (18)
56–65	77 (21)	72 (21)
Above 65	93 (25)	141 (42)
**Gender**		
Female	186 (51)	261 (77)
Male	181 (49)	77 (23)
**Highest level of education**		
Primary	97 (26)	68 (20)
Secondary	81 (22)	84 (25)
Tertiary	100 (28)	163 (48)
No formal education	89 (24)	23 (7)
**Marital status**		
Married	87 (24)	87 (26)
Divorced	96 (26)	91 (27)
Single	96 (26)	84 (25)
Widowed	88 (24)	76 (22)
**Current employment status**		
Employed	173 (47)	94 (28)
Retired	30 (9)	174 (51)
Self-employed	76 (20)	0 (0)
Unemployed	88 (24)	70 (21)

### Clinical characteristics of patients

At the Akoma Pa sites, 62% of patients were diagnosed with hypertension alone, while 5% had diabetes only, and 3% had both conditions. In contrast, the comparator group had 45% hypertensives, 7% diabetics, and 35% with both conditions. All patients in the Akoma Pa group received tele-counselling, prescription refill reminders, and medication adherence counselling, which were absent in the comparator group. Additionally, 82% of Akoma Pa patients participated in patient support groups (see Table 3).

**Table 3:** Clinical characteristics of patients

Variable	Akoma Pa (*n* = 367)	Conventional (*n* = 338)
**Health condition**		
*Hypertensives only*	229	174
*Diabetics only*	18	29
*Both Hypertension and Diabetes*	12	135
**Received Tele-counselling**	367	0
**Prescription Refill reminders**	367	0
**Medication Adherence Counselling**	367	0
**Patient support group**	367	0

### Comparative cost analysis

Table 4 presents the aggregate and disaggregated costs for both arms. The analysis reveals that the intervention achieved a lower total annual cost despite the addition of technology and program staff.

**Table 4:** Comparative annual costs (USD)

Cost category	Standard care (Comparator)	Akoma Pa (Intervention)
Consumables (recurrent)	$233,841 (High purchasing of test strips/reagents)	$114,790 (Data-driven purchasing)
Capital/equipment	$11,874 (Desktop computers, servers)	$6,326 (Tablets, phones, BP monitors)
Personnel	**$279**,**190** *(Facility staff only)*	**$363**,**144** *(Incl. Medtronic program staff)*
Training/capacity building	**$581**	**$11**,**989**
Governance/operations	**$4**,**396**	**$12**,**194**
*Total annual cost*	***$529***,***882.28***	***$508***,***443.89***
Cost difference	**$21**,**438.39** (s*avings*)	

### Analysis of consequences

#### Clinical effectiveness

The Akoma Pa intervention significantly improved outcomes of diabetes and hypertension management. Respondents achieved notable reductions in HbA1c levels (mean reduction of 2.10% vs. 1.68% in the comparator group) and fasting blood glucose (mean reduction of 2.21 mmol/L vs. 1.89 mmol/L). Systolic blood pressure reduction was also greater in the intervention group (54.38 mmHg vs. 51.88 mmHg). The intervention group showed a higher percentage of patients with controlled hypertension (58.9% vs. 23.2% in the comparator group) and better glycaemic control (85.4% vs. 31.7%) (see Table 5).

**Table 5:** Health effects per health outcome (12 months follow-up period)

Health outcome	Intervention group	Comparator group
Mean systolic reduction	54.38mmHg	51.88mmHg
Mean fasting blood sugar reduction	2.21mmol/L	1.89mmol/L
Mean HbA1c reduction	2.10%	1.68%
Mean BMI reduction	4.42 kg/m2	0.61 kg/m2
Patients followed up (%)	89%	36.5%
Diastolic blood pressure reduction	0mmHg	27.1mmHg

#### Process outcomes (follow-up rates)

**Fig. 1:**
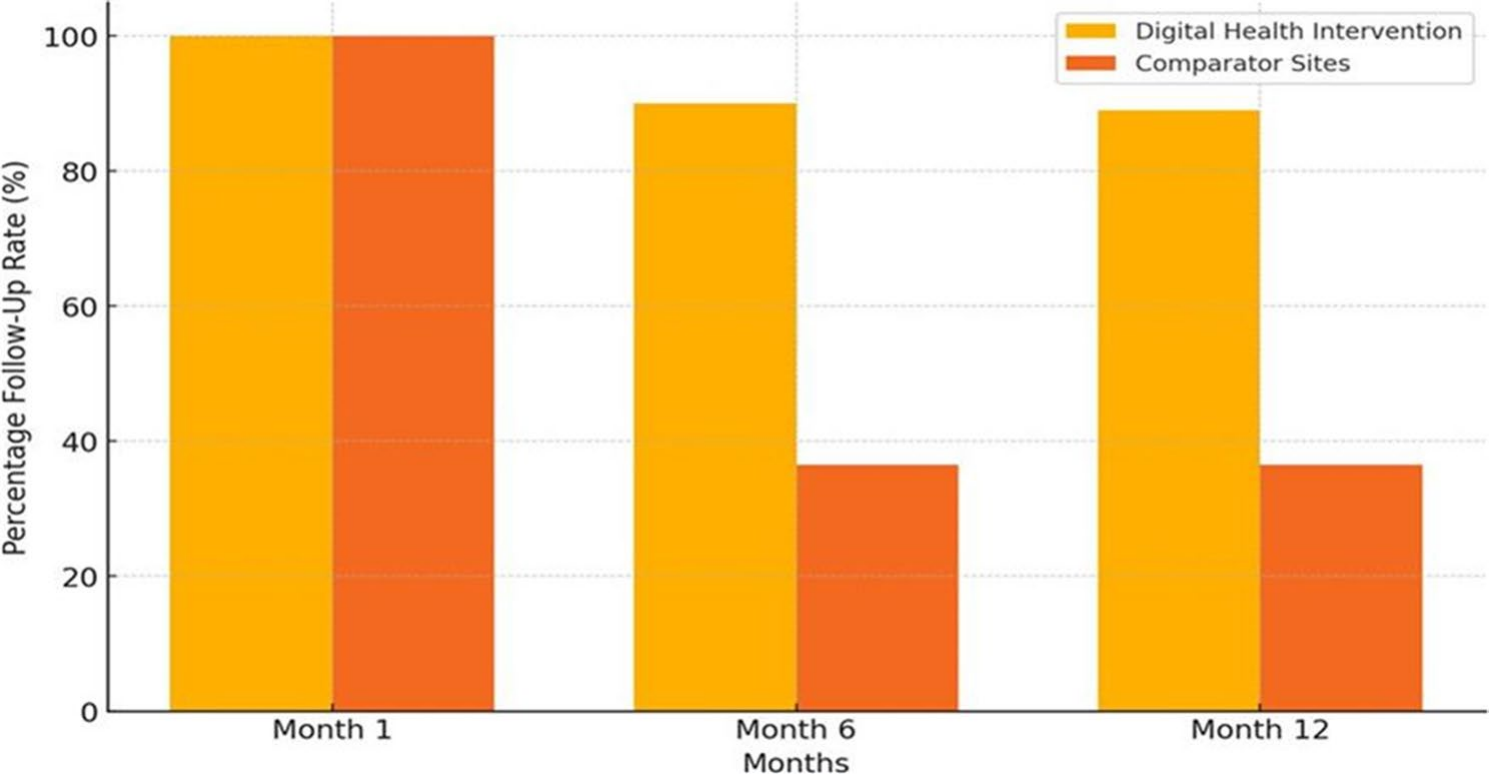
Monthly follow-up rates comparison

The Akoma Pa intervention significantly improved patient follow-up rates, 89% of patients in the intervention group completed follow-up visits, compared to 36.5% in the comparator group. This improvement was attributed to the use of digital health tools, including tele-counselling and automated reminders, which enhanced patient engagement and adherence to treatment plans (see Fig. 1).

### Cost-consequence balance sheet

The cost-consequence balance sheet (Table 6) synthesizes the findings, allowing for a direct comparison of inputs and outputs.

**Table 6:** Cost-Consequence Balance Sheet (Costs in USD)

Domain	Metric	Standard Care (Comparator)	Akoma Pa (Intervention)	Difference
Direct medical	*Consumables/ medications*	$233,841	$114,790	**$119**,**051** *(savings)*
Direct non-medical	*Training/capacity building*	$581	$11,989	**-$11**,**408** *(investment)*
Personnel	*Staff salaries*	$279,190	$363,144	**-$83**,**954** *(investment)*
Capital	*Technology/hardware*	$11,874	$6,326	**$5**,**548** *(savings)*
**Total cost**	*Annual program cost*	**$529**,**882**	**$508**,**444**	**$21**,**438** *(net saving)*
**Consequences**				
Clinical effectiveness *(mean)*	*SBP reduction*	-51.88 mmHg	-54.38 mmHg	**-2.50 mmHg**
	*HbA1c reduction*	-1.68%	-2.10%	**-0.42%**
	*FBS reduction*	-1.89 mmol/L	-2.21 mmol/L	**-0.32 mmol/L**
	*BMI reduction*	-0.61 kg/m²	-4.42 kg/m²	**-3.81 kg/m²**
Process	*Follow-up rate*	**36.5%**	**89%**	**+ 52.5%**
Disease control *(controlled rate %)*	*Hypertension*	23.2%	58.9%	**+ 35.7%**
	*Diabetes*	31.7%	85.4%	**+ 53.7%**

### Sensitivity analysis on cost and health outcomes

**Fig. 2:**
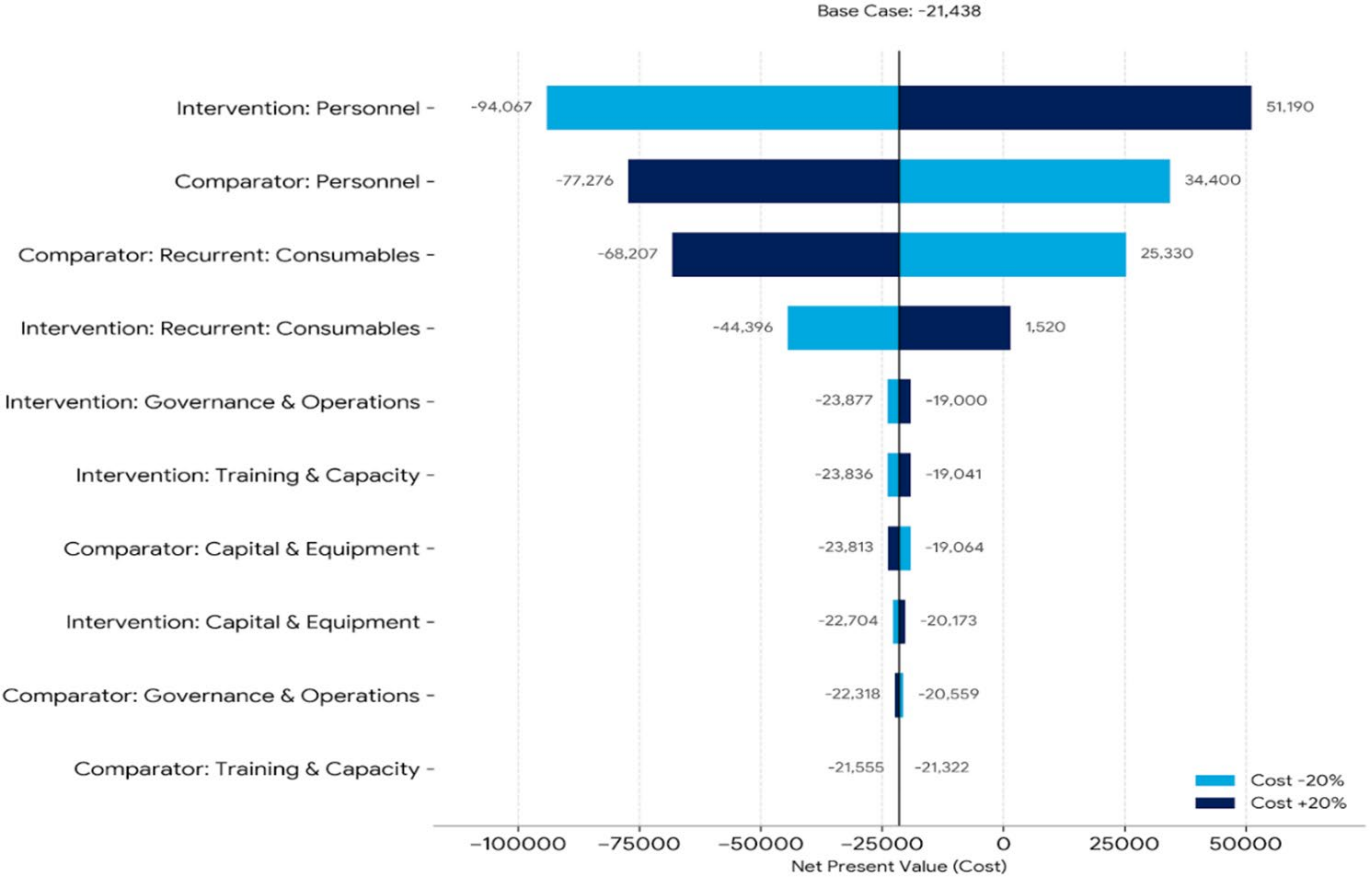
One-way sensitivity analysis cost

The one-way sensitivity analysis demonstrated that the net present value (NPV) was most sensitive to variations in personnel costs, particularly within the intervention facilities. A ± 20% change in intervention personnel costs shifted the NPV from − 94,067 to 51,190, indicating that personnel assumptions are the primary drivers of economic uncertainty. Comparator personnel costs and recurrent consumables also showed substantial influence. In contrast, variations in training, governance, and capital equipment costs had minimal impact on the overall results (see Fig. 2). This indicates that the economic conclusions are robust to most cost inputs but highly dependent on personnel cost estimates.

**Fig. 3:**
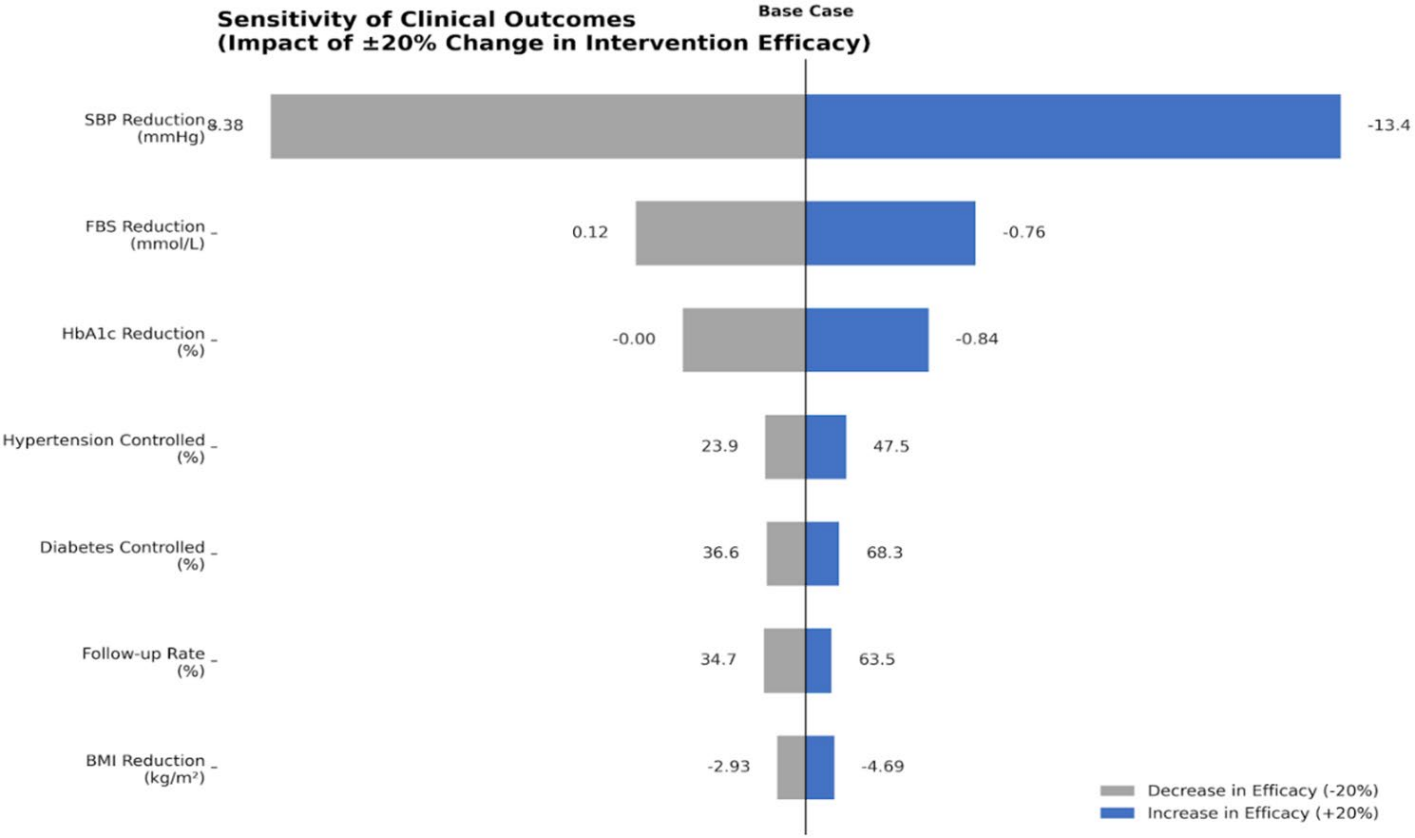
One-way sensitivity analysis on health outcomes

Figure 3 summarizes a one-way sensitivity analysis on health outcomes for a diabetes/hypertension management intervention under three scenarios. In the base case, modest improvements are seen, such as reduction in systolic blood pressure and decrease in BMI, though fasting blood sugar and HbA1c show minimal change. A 20% decrease in intervention efficacy leads to negative effects, including increases in fasting blood sugar and HbA1c. Conversely, a 20% increase in efficacy substantially improves outcomes, nearly doubling hypertension and diabetes control rates, increasing follow-up adherence, and amplifying BMI reduction. These highlight modest variations in program effectiveness and can significantly impact population-level health metrics.

## Discussion

This study evaluated the cost-consequence of the Akoma Pa intervention designed to improve the management of hypertension and diabetes in Ghana. Akoma Pa delivered better clinical outcomes and patient follow-up while costing the health system less overall than standard care. Despite higher spending on staff and training, the program reduced total annual costs through lower consumable and capital expenses enabled by digital and data-driven processes. Patients in the intervention arm experienced greater improvements in blood pressure, blood sugar, HbA1c, and BMI. Also, higher disease control and follow-up rates demonstrate that the digitally enabled model achieved better results at a lower total cost. The findings offer valuable insights for scaling digital health interventions in LMICs, with implications for global NCD management strategies.

The cost-consequence analysis of the Akoma Pa program demonstrates important economic and clinical advantages that are particularly relevant in resource-limited settings. When costs and outcomes are examined side by side, the intervention achieves greater reductions in systolic blood pressure while operating at a lower total annual cost than standard care. This indicates that improved blood pressure control was attained without additional financial burden to the health system. The findings highlight the program’s dual benefit: it enhances clinical outcomes while reducing overall resource use, likely lowering the need for more expensive downstream care such as hospitalizations and emergency services. This finding aligns with global evidence demonstrating the potential of digital health interventions to enhance hypertension management while optimizing resource allocation in healthcare systems [23].

Similarly, the reduction in HbA1c highlights the intervention’s role in improving diabetes management. HbA1c, a key indicator of long-term blood glucose control, is critical for preventing diabetes-related complications. The substantial reduction in HbA1c at a lower cost suggests that the Akoma Pa intervention facilitates more effective diabetes management, potentially reducing reliance on costly medications and interventions for complications. This finding is consistent with other studies demonstrating the therapeutic effectiveness of digital health solutions in managing diabetes [24–26]. Furthermore, economic evaluations of digital health interventions, including telemonitoring, SMS-based interventions, and remote consultations, have consistently shown positive impacts on both clinical outcomes and cost savings [27, 28]. These results underscore the potential of digital health interventions to address the growing burden of diabetes in LMICs, where access to specialized care is often limited.

Reduction in BMI introduces another critical dimension to the program’s impact. Given the high comorbidity of obesity with both diabetes and hypertension, effective weight management is essential for improving overall health outcomes. This finding is supported by a growing body of evidence demonstrating the positive effects of telemonitoring and tele-screening on glycaemic control, weight management, and physical activity [29–31]. These results highlight the potential of digital health interventions to address multiple risk factors simultaneously, offering a holistic approach to NCD management.

The findings on patient follow-up further underscore the program’s ability to sustain long-term patient engagement, a critical factor for the successful management of chronic conditions. The Akoma Pa intervention not only supports continuous patient interaction but also offers substantial economic advantages by reducing healthcare costs. This finding is particularly relevant in LMICs, where patient retention and adherence to treatment plans are often challenging due to financial, geographic, and systemic barriers. Previous studies have consistently highlighted the therapeutic benefits of digital health interventions in improving patient adherence and health outcomes, ultimately contributing to better disease management and reduced long-term healthcare expenditures [24–26].

The findings of this study have significant implications for global health, particularly in LMICs grappling with the dual burden of infectious diseases and NCDs. The Akoma Pa program’s cost-consequence and clinical effectiveness demonstrate that scaling up digital health interventions could alleviate the economic burden of NCDs while improving patient outcomes. Policymakers in LMICs should consider integrating digital health solutions into national NCD management strategies, particularly in settings with limited healthcare resources. Investments in digital infrastructure, capacity building for healthcare providers, and patient education could further enhance the impact of such interventions. Moreover, the success of the Akoma Pa program offers a replicable model for other LMICs facing similar challenges in NCD management. By leveraging widely accessible technologies such as mobile phones and telemonitoring, digital health interventions can bridge gaps in healthcare delivery, particularly in rural and underserved areas. Global health organizations and funding agencies should prioritize support for the development and implementation of digital health solutions as part of comprehensive NCD control strategies.

### Strengths and limitations

This study offers valuable evidence on the effectiveness and cost-consequences of digital health interventions for chronic disease management. The study’s non-randomized design may have introduced selection bias and unmeasured confounding, limiting causal inference. The short follow-up period likely underestimates the long-term health and economic benefits of improved chronic disease control while capturing most implementation costs. Additionally, the findings may have limited generalizability beyond the study facilities, as variations in staffing, resource availability, and health system context, particularly personnel costs, could influence results in other settings. Despite these limitations, the study provides important evidence on the costs and multiple health and service delivery consequences associated with digital health interventions for chronic disease management.

## Conclusion

The Akoma Pa program demonstrates that digital health interventions can enhance NCD management in Ghana by improving clinical outcomes and patient retention while simultaneously reducing overall health system costs. The cost-consequence balance sheet reveals that investments in training and personnel are fully offset by savings in medical consumables and capital equipment. These findings support the scaling of digitally enabled care models as a sustainable strategy for NCD management in resource-limited settings.

## Data Availability

All data generated or analysed during this study are included in this article.

## Declarations

### Approach to engagement

The study engaged patients, healthcare providers, and stakeholders in the design and implementation of the Akoma Pa program. Patient support groups and tele-counselling sessions facilitated direct engagement with patients, while training programs for healthcare professionals ensured their involvement in the intervention. Feedback from these groups informed the program’s design and evaluation.

## Abbreviations

BMI: Body mass index
CCA: Cost-consequences analysis
CHAG: Christian health association of Ghana
FBS: Fasting blood sugar
ICER: Incremental cost-effectiveness ratio
LMICs: Low-and Middle-Income Countries
NCDs: Non-communicable diseases
NPV: Net present value
SBP: Systolic blood pressure
SDGs: Sustainable Development goals
WHO: World Health Organization
